# Impacts of obesity, maternal obesity and nicotinamide mononucleotide supplementation on sperm quality in mice

**DOI:** 10.1530/REP-18-0574

**Published:** 2019-05-30

**Authors:** Neil A Youngson, G Mezbah Uddin, Abhirup Das, Carl Martinez, Haley S Connaughton, Sara Whiting, Josephine Yu, David A Sinclair, R John Aitken, Margaret J Morris

**Affiliations:** 1 Department of Pharmacology, School of Medical Sciences, University of New South Wales, Sydney, New South Wales, Australia; 2 Paul F. Glenn Center for the Biological Mechanisms of Aging, Department of Genetics, Blavatnik Institute,Harvard Medical School, Boston, Massachusetts, USA; 3 School of Environmental and Life Sciences, University of Newcastle, Callaghan, New South Wales, Australia

## Abstract

Male fertility and sperm quality are negatively impacted by obesity. Furthermore, recent evidence has shown that male offspring from obese rat mothers also have reduced sperm quality and fertility. Here, we extend work in this area by comparing the effects of both maternal obesity and offspring post-weaning diet-induced obesity, as well as their combination, on sperm quality in mice. We additionally tested whether administration of the NAD^+^-booster nicotinamide mononucleotide (NMN) can ameliorate the negative effects of obesity and maternal obesity on sperm quality. We previously showed that intraperitoneal (i.p.) injection of NMN can reduce the metabolic deficits induced by maternal obesity or post-weaning dietary obesity in mice. In this study, female mice were fed a high-fat diet (HFD) for 6 weeks until they were 18% heavier than a control diet group. Thereafter, HFD and control female mice were mated with control diet males, and male offspring were weaned into groups receiving control or HFD. At 30 weeks of age, mice received 500 mg/kg body weight NMN or vehicle PBS i.p. for 21 days. As expected, adiposity was increased by both maternal and post-weaning HFD but reduced by NMN supplementation. Post-weaning HFD reduced sperm count and motility, while maternal HFD increased offspring sperm DNA fragmentation and levels of aberrant sperm chromatin. There was no evidence that the combination of post-weaning and maternal HFD exacerbated the impacts in sperm quality suggesting that they impact spermatogenesis through different mechanisms. Surprisingly NMN reduced sperm count, vitality and increased sperm oxidative DNA damage, which was associated with increased NAD^+^ in testes. A subsequent experiment using oral NMN at 400 mg/kg body weight was not associated with reduced sperm viability, oxidative stress, mitochondrial dysfunction or increased NAD^+^ in testes, suggesting that the negative impacts on sperm could be dependent on dose or mode of administration.

## Introduction

Obesity can severely impact fertility and reproductive organ function (Cardozo *et al.* 2011, Liu & Ding 2017). Human studies have shown that BMI inversely correlates with sperm count and various measurements of sperm quality including sperm motility, morphology and DNA damage (Jensen *et al.* 2004, Chavarro *et al.* 2010). Obesity reduces sperm quality through various means. These include changes in hormone levels such as leptin, testosterone and oestrogen (Considine *et al.* 1996, Chavarro *et al.* 2010) and an increase in the localised and systemic levels of oxidative stress (Gavriliouk & Aitken 2015, Liu & Ding 2017). Excess fat deposits have been shown to increase scrotal temperature in humans, which is thought to reduce sperm quality by denaturing enzymes that are required for spermatogenesis (Hjollund *et al.* 2000, Kort *et al.* 2006). In both humans and mice, obesity promotes the aromatisation of testosterone to estradiol (E2), which is known to impair spermatogenesis by reducing the level of testosterone in the body (Hjollund *et al.* 2000, Erdemir *et al.* 2012). In addition to E2, higher BMI was also found to reduce the levels of reproductive hormones such as inhibin B, follicle-stimulating hormone and luteinising hormone (Chavarro *et al.* 2010), which all play essential roles in spermatogenesis. Evidence from mice has also shown that obesity disrupts the blood–testis barrier, which may expose both the spermatogenic cells to blood-borne noxious agents and the seminiferous epithelium to autoimmune events (Fan *et al.* 2015). The correlation between sperm DNA damage and increased BMI (Kort *et al.* 2006, Chavarro *et al.* 2010, Dupont *et al.* 2013, Gavriliouk & Aitken 2015) is thought to be linked to increased oxidative stress. While spermatozoa contain antioxidant enzymes, increased reactive oxygen species (ROS) levels in obesity are known to cause DNA damage and initiate apoptosis among spermatozoa, thereby decreasing sperm concentrations and increasing the likelihood of male infertility or failed pregnancies (Moustafa *et al.* 2004, Agarwal *et al.* 2014, Gavriliouk & Aitken 2015).

A feature of the worldwide increase in obesity rates (World Health Organisation 2015) is the corresponding increase in maternal obesity, that is in women prior to and during pregnancy and lactation (Callaway *et al.* 2006, Reynolds *et al.* 2013). Maternal obesity is linked to increased offspring birth weight, adiposity (Martin *et al.* 2015, Starling *et al.* 2015) and a higher risk of pregnancy complications (Callaway *et al.* 2006, Avci *et al.* 2015, Sullivan *et al.* 2015). Postnatally, offspring from obese mothers have reduced survival rate (Reynolds *et al.* 2013), which is linked to increased risk for diseases such as CVD (Reynolds *et al.* 2013), metabolic syndrome (Boney *et al.* 2005) and non-alcoholic fatty liver disease (Oben *et al.* 2010). Maternal obesity can also reduce fertility in both male (Rodriguez-Gonzalez *et al.* 2015, Santos *et al.* 2015) and female (Ambrosetti *et al.* 2016, Tsoulis *et al.* 2016) offspring. Studies in rat have found oxidative stress levels to be elevated in the testis and sperm of offspring of obese mothers (Rodriguez-Gonzalez *et al.* 2015, Santos *et al.* 2015). Therefore, maternal obesity has the potential to not only negatively impact offspring health, but the health of grand-offspring via alteration of offspring sperm DNA or epigenetic state (Ge *et al.* 2014, Rodriguez-Gonzalez *et al.* 2015, Santos *et al.* 2015, Soubry *et al.* 2015). An additional consequence of maternal obesity is the exacerbation of negative health impacts as the offspring themselves are at a higher risk of becoming obese (Bahari *et al.* 2013, Uddin *et al.* 2017). This effect is driven by developmental programming of offspring metabolism and can lead to a more severe phenotype than ‘first-generation obesity’ (Starling *et al.* 2015, Elshenawy & Simmons 2016).

NAD^+^ is a key metabolite, which also acts as a central signalling molecule and enzyme substrate with numerous roles in fundamental biological processes including energy metabolism, lifespan regulation, DNA repair, apoptosis and telomere maintenance (Yang & Sauve 2016). Boosting NAD^+^ levels through exercise (Uddin *et al.* 2016, 2017), supplementation with NAD^+^ boosters, (usually precursor molecules) (Yoshino *et al.* 2011, Gariani *et al.* 2016, Uddin *et al.* 2016, Das *et al.* 2018) or increasing the activity of NAD^+^-dependent effectors (e.g. Sirtuin proteins) (Baur *et al.* 2006) have all been used effectively to recapitulate exercise-like benefits in obese and aged rodent models. We previously examined the effects of administration of the NAD^+^-booster nicotinamide mononucleotide (NMN) in maternal obesity by supplementing female offspring. We found improvements in offspring adiposity, metabolism (Uddin *et al.* 2017) and the reversal of some changes in markers of oocyte-secreted factor signalling (Bertoldo *et al.* 2018).

The effects of maternal obesity on offspring sperm quality are poorly understood. Considering the impact of the worldwide obesity epidemic on disease risk as well as fertility, there is a need to investigate this area more. Here, we add to the knowledge on how maternal obesity affects sperm quality in mice, as well as provide the first information on how NMN supplementation affects sperm quality in the context of normal weight, as well as obesity associated with both post-weaning and maternal HFD consumption. We hypothesised that similar to improvements in body-wide and liver metabolism, sperm quality would benefit from NMN supplementation in obese mice. However, our results suggest that in some circumstances NMN supplementation may actually be detrimental to sperm quality.

## Materials and methods

### Animal procedures

The effects of NMN on sperm was analysed in two mouse cohorts. One cohort investigated the effects of daily intraperitoneal (i.p.) injections of NMN on sperm from post-weaning HFD-induced obese mice and mice from a mother fed either a chow diet or a HFD. The second cohort investigated the effects of NMN administered in drinking water on sperm from chow-fed animals only, with experiments on live sperm focussing on measuring oxidative stress and mitochondrial function.

#### Maternal obesity cohort design

Female C57BL/6 mice (Animal Resource Centre, Canning Vale, WA, Australia) were housed in the Biological Resource Centre at 21°C ± 2, with a 12 h/12 h light cycle. Mice were housed four per cage and fed standard chow pellets (11 kJ/g, 4% total fat, 13% digestible energy from lipids, Gordon’s Stock Feeds, Yanderra, NSW, Australia). At 4 weeks of age, females were separated into high-fat and control chow diets. High-fat diet (HFD) was 19 kJ/g, composed of 23.5% total fat, 43% digestible energy from lipids (Specialty Feeds, Glen Forrest, WA, Australia). When HFD-fed females weighed 18% more than control mice (between 9 and 10 weeks), they were mated with males (same provider) fed with standard show diet (four females and one male per cage). To control for parity effects only virgin females were bred. Due to the expected low pregnancy rates and high levels of cannibalism in first-litters (especially in HFD-fed females), and the use of inexperienced virgin males, we started with 48 chow-fed and 80 HFD-fed females. Pregnancy was confirmed by body weight gain 1 week post mating, after which dams were housed one per cage and maintained on their pre-pregnancy diet throughout pregnancy and lactation. Dams gave birth at approximately 12–13 weeks of age. All pups were born over a 3-day period and were left undisturbed for the first week to prevent maternal stress. At PND 24, each mouse received an ear punch. For all molecular analyses, the use of this individual identifier ensured that experimenters were blind to treatment group of the animals.

At PND 28 male offspring were weaned and distributed across chow or HFD groups, ensuring that the group average body weights were as near to equivalent as possible (with the only differences being due to the dam’s diet, that is all group average weights were larger if the dam was fed HFD). Siblings were split between groups as often as possible to control for litter-effects which resulted in the 10–12 pups in each group being from a minimum of six dams.

**Figure 1:**
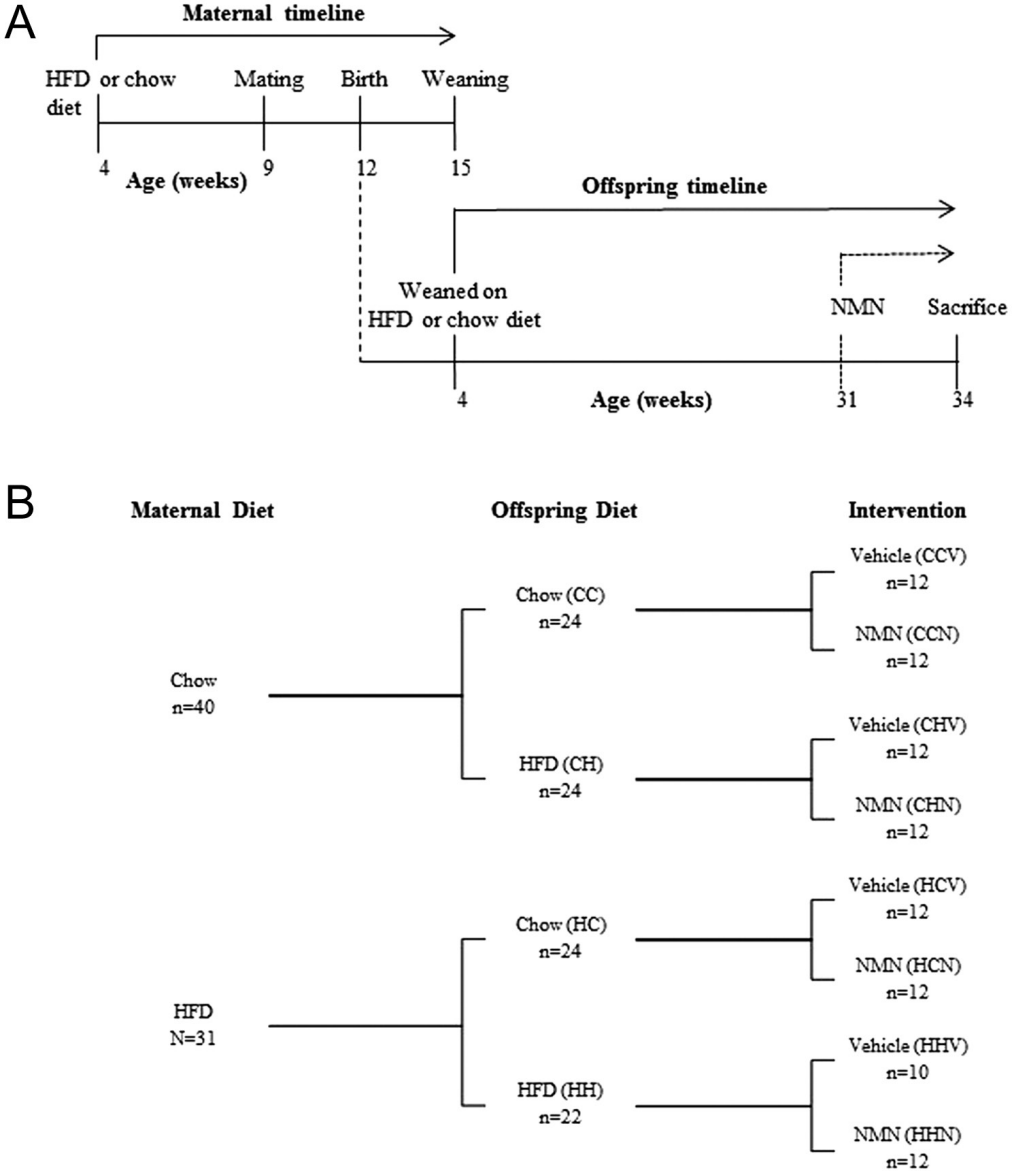
Offspring timeline and maternal obesity cohort design. (A) Male offspring of mothers were weaned on HFD or chow diet at 4 weeks of age until cull (33 weeks). At 31 weeks of age, a subgroup of offspring from both maternal diet groups was injected (i.p.) with NMN daily. (B) Mothers were fed with either chow diet (C) or HFD (H). Offspring of both maternal groups were fed with either chow diet (CC and HC) or HFD (CH and HH). Nicotinamide mononucleotide (NMN) was injected, intraperitoneally, in four groups (CCN, CHN, HCN and HHN). Phosphate buffered saline vehicle was injected in the other four groups (CCV, CHV, HCV, HHV).

Pups were weaned onto either HFD or control diet; diets were maintained through to 34 weeks of age. A subgroup of offspring from both maternal diet groups was given daily i.p. injections of NMN (500 mg/kg body weight) from 31 to 34 weeks of age. All non-NMN-treated offspring received daily injection of PBS vehicle. Each group containing 10–12 offspring were killed at 34 weeks. The experimental timeline and groups are illustrated in Fig. 1A and B, respectively. Tissues of interest were harvested at sacrifice and snap-frozen in liquid nitrogen and stored at −80°C. All animal procedures were approved by Animal Ethics Committee, UNSW (Ethics number 13/25B).

#### Oral administration of NMN cohort design

Two-month-old male C57BL/6 mice were housed in the Biological Resource Centre at UNSW and fed the standard chow diet. Half of the cohort was given NMN (400 mg/kg body weight) via drinking water for 24 weeks; controls received drinking water. At 6 months, mice were killed and tissue was collected. All animal procedures were approved by Animal Ethics Committee, UNSW (ACEC 16/13B).

#### Sperm collection

Sperm was collected from vas deferentia and cauda epididymides of mice into 1 mL of DMEM or, if FACS was to be performed in 1 mL of Biggers, Whitten and Whittingham media (BWW). Motility, vitality and concentration were assessed under a light microscope using a haemocytometer. To examine sperm motility, 5 μL sperm suspension were added to 45 μL BWW working solution. For sperm count, 5 μL sperm suspension were added to 45 μL sperm diluting fluid (2.5 g NaHCO_3_, 0.5 mL formaldehyde and 49.5 mL in water). For vitality 5 μL sperm suspension were added to 5 μL eosin stain (0.5% w/v eosin, 0.9% w/v NaCl in water) and immediately examined by haemocytometer. Sperm with pink heads were considered to be non-viable. Concurrent with the vitality measurement, sperm motility was assessed by counting the percentage of sperm that were moving (either slowly or vigorously). For FACS assays viability was measured with 7-Aminoactinomycin D (7-AAD). Aliquots containing a million sperm (in approximately 200 μL DMEM) were snap-frozen in liquid nitrogen and stored for 8-hydroxy-2′-deoxyguanosine (8-OHdG), halo, chromomycin A3 (CMA3) and sperm chromatin structure assay (SCSA) assays. For FACS experiments aliquots of the sperm suspension were further diluted in BWW to make multiple 190 μL aliquots of 10^6^/mL sperm for staining.

#### Frozen sperm assays

Oxidative DNA damage (8-OHdG) immunofluorescence (Houston *et al.* 2018) and CMA3 staining (Smith *et al.* 2013) were performed with published laboratory protocols. For 8-OHdG measurement, spermatozoa were snap-frozen in liquid nitrogen and stored at −80°C until analyses could occur. These cells were then resuspended in primary DNA/RNA damage antibody (Novus Biologicals) (25 μg/mL in PBST) overnight at 4°C. After primary incubation, spermatozoa were washed in PBS and then incubated with Alexa Fluor 488 goat α mouse secondary (5 μg/mL in PBST) for 1 h at 37°C. Cells were washed twice in PBS and viewed by fluorescence microscopy. A sample of 100 cells was assessed, scoring positive by the presence of nuclear fluorescence. 8-OHdG was chosen for as a marker for sperm oxidation as it has been used as such in human (Vorilhon *et al.* 2018) and rodent (Kocer *et al.* 2015) studies of environmentally induced (Ahmed-Farid *et al.* 2017) and genetically induced (Huang *et al.* 2016) sperm DNA damage.

For CMA3 measurement spermatozoa were frozen in liquid nitrogen and thawed at room temperature. Cells were centrifuged and resuspended cells into McIlvaine buffer (17 mL of 0.1 mol/L citric acid mixed with 83 mL of 0.2 mol/L Na_2_HPO_4_ and 10 mmol/L MgCl_2_, pH 7.0). The chromatin of sperm cells were labelled by incubating with 25 µL of CMA3 solution (0.25 mg/mL in McIlvaine buffer) for 20 min in the dark at room temperature. Cells were washed with McIlvaine buffer before counting labelled cells under fluorescent microscope.

The Sperm Chromatin Dispersion Test (halo) assay was a modification of a previously published protocol (Fernandez *et al.* 2003). Briefly, spermatozoa were frozen in liquid nitrogen and thawed at room temperature. Cells were mixed with 1% low melting agarose to a final concentration of agarose 0.7%, at 37°C. The cell–agarose mixture was put on to a slide pre-coated with 0.65% standard agarose. Slides were solidified at 4°C for 4 min. Coverslip was carefully removed and placed horizontally in acid denaturation solution (0.08 N HCl) for 7 min at room temperature. Slides are then incubated in a neutralising and lysing solution 1 (0.4 M Tris, 0.8 M DTT, 1% SDS and 50 mM EDTA, pH 7) for 10 min at room temperature, then into neutralising and lysing solution 2 (0.4 M Tris, 2 M NaCl and 1% SDS, pH 7.5) for 5 min at room temperature. Slides are then washed in Tris-borate-EDTA buffer (0.09 M Tris-borate and 0.002 M EDTA, pH 7.5) for 2 min, dehydrated in 70, 90 and 100% ethanol for 2 min each at room temperature and air dried. Slides were stained with DAPI for 10 min at room temperature. Slides were counted under a fluorescent microscope for ‘halo’ or ‘no halo’.

The SCSA performed as described previously with modification (Evenson & Jost 2000). Briefly, spermatozoa were snap-frozen in liquid nitrogen and stored at −80°C until further analysis. The spermatozoa were thawed and placed on ice. Hundred microlitres of the sperm suspension was added to a FACS tube with 200 µL of acid detergent solution. After exactly 30 s, spermatozoa were stained with 600 µL of acridine orange staining solution. Using a FACScanTM Flow Cytometer (BD) debris was gated out and 5000 sperm events were acquired per sample. The ratio of single stranded (red) to double stranded (green) fluorescence (%DFI) was calculated using CellQuestTM software (BD).

#### Mitochondrial DNA (mtDNA) copy number measurement

This was performed by qPCR on isolated sperm DNA with primers for mtDNA in the *Cytb* gene with normalisation to the nuclear genome in the *Rplp0* gene as previously described (Uddin *et al.* 2016).

#### Live sperm FACS staining

Samples from each mouse were assessed for viability, mitochondrial membrane potential (MMP, ΨM%), and oxidative stress using the following dyes: 7-Aminoactinomycin D (7-AAD) (BD Biosciences), JC-1 dye (Life Technologies) and MitoSOX Red (MSR) Mitochondrial Superoxide Indicator (Life Technologies). The component of each tube used to examine MMP and oxidative stress are as described in Supplementary Table 1 (see section on supplementary data given at the end of this article). All tubes were incubated at 37°C (5% CO_2_) for 15 min then centrifuged for 3 min at room temperature, 1500 ***g***. The supernatant was aspirated and pellets were redissolved in 400 μL of BWW working solution. Samples were kept on ice before evaluation by flow cytometry 10 min later. The time from removal of cauda epididymis from the mouse until initiation of FACS analysis was approximately 30 min.

#### Flow cytometry

Flow cytometry was conducted at the Biological Resource Imaging Laboratory (BRIL) of the Mark Wainwright Analytical Centre (UNSW, Australia). Samples were evaluated using a BD FACSVerse™ flow cytometer (BD Biosciences, CA, USA) and acquisitions were obtained using the BD FACSuite™ software (BD Biosciences). Dyes were excited by a 488 nm laser. Overall, 50,000 events were acquired for each sample. Data were analysed using the FlowJo v10.2 software (FlowJO LLC, Oregon, USA). A forward-scatter and side-scatter cytogram were used to identify and gate for sperm cells as described in a previous study (Yeung *et al.* 2002) (Supplementary Fig. 1A). From this gated population, viable sperm cells were identified as 7-AAD negative (7-AAD –ve; emission wavelength 647 nm) and gated for subsequent JC-1 or MSR analyses (Supplementary Fig. 1B and C). Results were expressed as the percentage of 7-AAD –ve sperm cells. Only gated viable sperm were examined for oxidative stress or MMP.

To examine oxidative stress levels, viable sperm cells were divided into two groups: those with low MSR fluorescence and with high MSR fluorescence, as detected with a phycoeryhtrin (PE) filter (emission wavelength 578 nm) which are representative of low and high oxidation, respectively (Supplementary Fig. 1D and E). Results were expressed as the percentage of live sperm cells positive for high MSR.

To examine MMP, the viable sperm cells were plotted on a fluorescein isothiocyanate (FITC; emission wavelength 519 nm) versus phycoerythrin (PE) cytogram (Supplementary Fig. 1F). FITC and PE measures the green and orange fluorescence emitted by JC-1, respectively. The cytogram was divided into quadrants (Supplementary Fig. 1F) as described by Binet *et al.* (2014). Results are expressed as the percentage of live sperm in the high orange (HO) Q1 or high green (HG) Q3 or the ratio between HO and HG fluorescence that is the ratio between Q1 + Q2 and Q2 + Q3, respectively.

#### Measurement of testes NAD^+^ and NADH levels

Testes were homogenised in extraction buffer (50 mM Tris HCl, 10 mM nicotinamide, 0.1% Triton X-100) and centrifuged (7000 ***g*** for 5 min at 4°C). An aliquot of the supernatant was taken for protein quantification. The remainder of the supernatant was passed through (14,000 ***g*** for 45 min at 4°C) a 10 kDa filter (Amicon) and flowthrough was spilt into two aliquots. One was placed on ice to measure total NAD while the other was used to determine NAD^+^ content through acidification and heat treatment before being placed back on ice. Samples were then reacted with alcohol dehydrogenase on a 96-well plate at room temperature, followed by quantification using the Bio-Rad iMark microplate reader. All steps apart from the filtering of lysate through a column were performed as previously described (Uddin *et al.* 2016).

### Statistical analyses

Maternal obesity cohort: GraphPad Prism v6.0 software (GraphPad Software) was used to assess the normality of the data using the D’Agostino-Pearson omnibus test. Statistical analyses were performed using IBM SPSS Statistics 22 (IBM Corp.). Three-way ANOVA was performed to examine the effects of maternal diet, offspring diet and intervention on phenotype measurements. Simple main effects were performed to examine any significant effect caused by an interaction between these factors. Post-hoc comparisons were Bonferroni corrected. Results are expressed as mean ± s.e.m. Results were considered significant if *P* ≤ 0.05.

Oral administration cohort: Two-way ANOVA was performed to analyse the effects of NMN on oxidative stress and MMP. Student’s *t*-test was used to examine the effects of NMN versus control on live sperm cohort. Results were considered significant if *P* ≤ 0.05.

## Results

### Maternal obesity cohort

#### Effects on body weights and offspring testes

**Figure 2:**
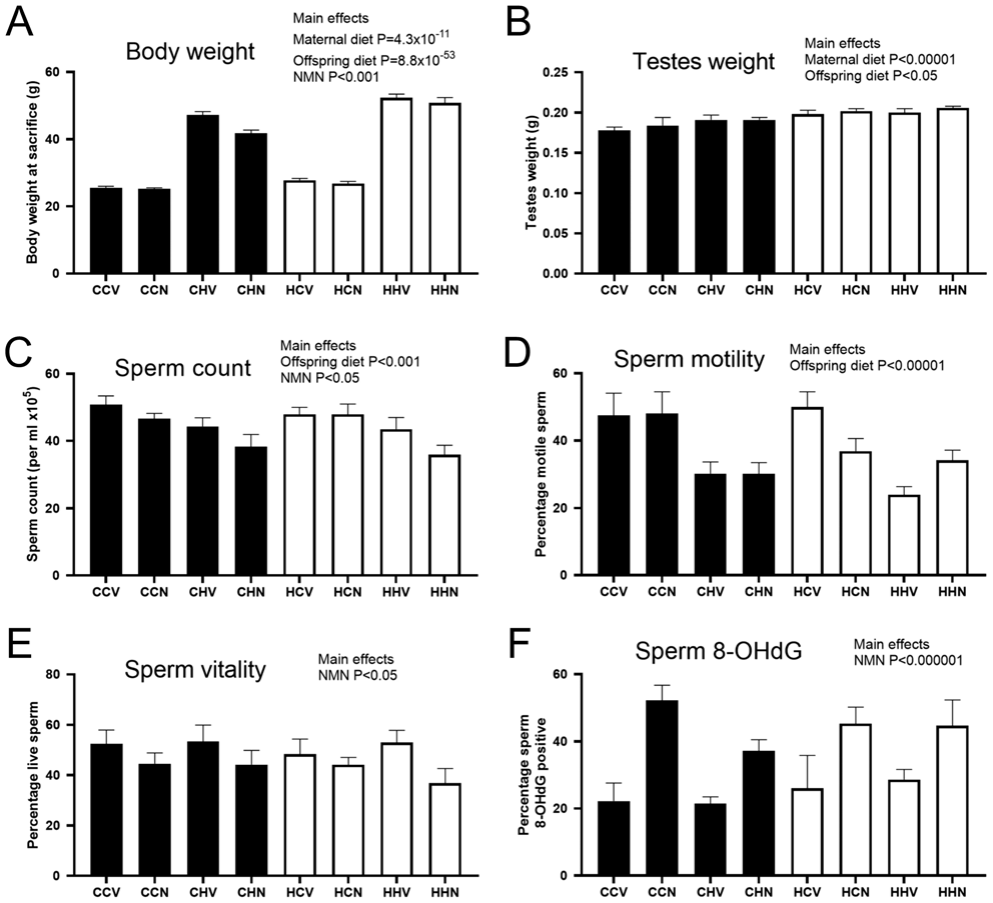
Effects of maternal obesity, offspring post-weaning diet and intraperitoneal NMN injection on anthropometric (A,B) and sperm parameters in offspring (C – F) in offspring. The first letter of the group code indicates maternal diet, chow (C) or HFD (H). The second letter indicates offspring post-weaning diet, chow (C) or HFD (H). The third letter indicates NMN (N) or vehicle (V) injection. Black bars indicate offspring from chow-fed mothers, white bars indicate offspring from HFD-fed mothers. *P* values from a three-way ANOVA that was used to compare all eight groups where maternal diet, post-weaning diet and treatment were three different factors. Data are mean ± s.e.m.

Prior to mating, after 6 weeks on the HFD, dams were 18% heavier than control dams (17 chow dams average 17.4 ± 1.0 g, 14 HFD dams average 20.7 ± 1.3 g, *P* = 7 × 10^−9^) due to an increase in adiposity (Uddin *et al.* 2017). Both maternal diet and offspring post-weaning HFD increased offspring body weight as expected (Fig. 2A). NMN supplementation reduced overall body weight (Fig. 2A). Adiposity, as determined by retroperitoneal fat pad mass at sacrifice followed the same trend, with increases due to post-weaning HFD (>500%), due to maternal obesity (>200%) and the combination of maternal obesity and post-weaning HFD (>700%) compared to chow-fed mice from chow-fed mothers, while NMN reduced adiposity in the HFD-fed mice (data in Uddin et al Manuscript Under Review). Both maternal and offspring post-weaning HFD increased testis weight (Fig. 2B). The maternal diet associated increase in testis weight was still significant when weight was divided by the length of the mouse; however, the post-weaning diet effect was lost (Supplementary Table 2).

#### Effects on live sperm measurements

Post-weaning HFD and NMN both reduced sperm count (Fig. 2C). Sperm motility was reduced by post-weaning HFD only (Fig. 2D). Sperm vitality was decreased by NMN treatment (where an eosin-stained head was considered to indicate a dead sperm) (Fig. 2E).

#### Effects on frozen sperm quality

NMN treatment had a striking effect on 8-OHdG levels in sperm, increasing it in all groups, compared to the corresponding control group (Fig. 2F). No significant effects were seen in the halo assay and variation was large in this measurement. CMA3 staining was increased by maternal diet (Supplementary Table 2), although the percentage of sperm that stained were low in all groups. In the SCSA, the %DFI was increased by maternal diet, and there was a trend for offspring HFD to increase it (Supplementary Table 2).

#### mtDNA copy number

Post-weaning HFD reduced mitochondrial copy number (Supplementary Table 2). The effects of NMN were more complex, as it increased copy number in offspring from chow-fed mothers but decreased it offspring from HFD mothers (Supplementary Table 2).

### Oral administration of NMN cohort

To further our investigation into the effects of NMN administration on sperm quality, we undertook a second cohort. This aimed to uncover whether NMN affected sperm when the drug is orally administered. Mice were maintained on chow diet with access to vehicle (water) or 400 mg/kg NMN for 24 weeks. There was no difference in the body weights of the 32-week-old males (Supplementary Table 3). Sperm count, motility and mitochondrial copy number also were not significantly different between the two groups (Supplementary Table 3).

#### FACS analysis of viability, oxidative stress and MMP in oral administration of NMN cohort

**Figure 3:**
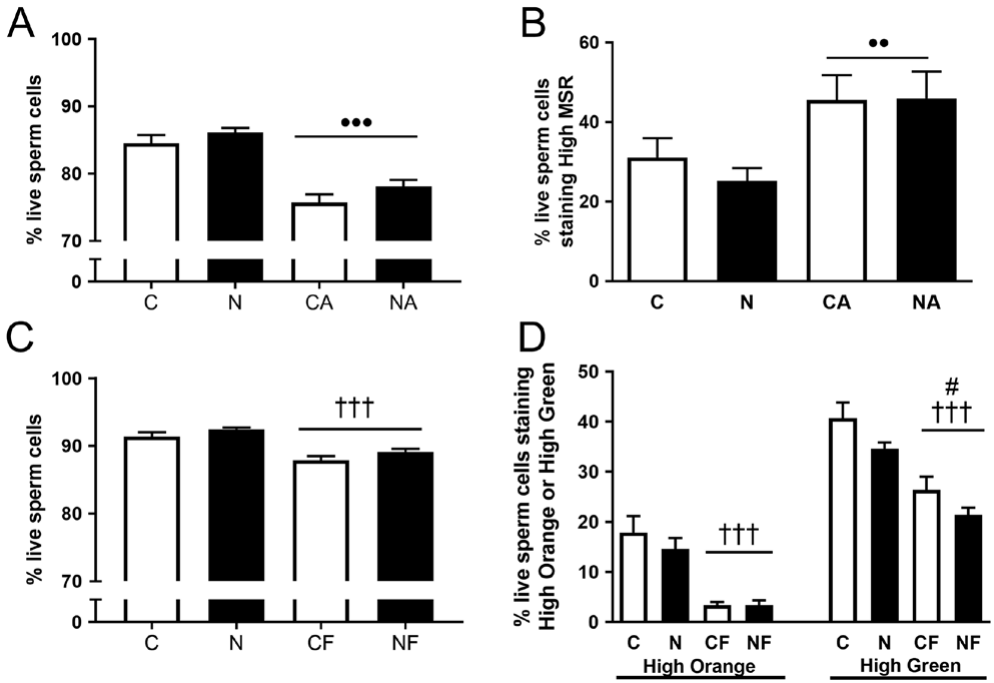
Viability, oxidative stress and mitochondrial membrane potential of sperm from control and NMN-treated mice in the Oral Administration Cohort. (A) Viability as determined by 7-AAD staining of sperm used for oxidative stress measurement. ^••^ *P* ≤ 0.01 reduction in viability due to positive control AA. (B) Oxidative stress as determined by mitosox red (MSR) staining. ^•••^ *P* ≤ 0.001 increase in oxidative stress due to positive control arachidonic acid (AA). (C) Viability as determined by 7-AAD staining of sperm used for mitochondrial membrane potential measurement. ^†††^ *P* ≤ 0.001 reduction in viability due to positive control FCCP. (D) Mitochondrial membrane potential as determined by JC-1 staining. ^†††^ *P* ≤ 0.001 reduction in HO and high green-staining sperm due to positive control FCCP. ^#^ *P* ≤ 0.05 reduction in high green-staining sperm due to NMN. C, Control mouse sperm; N, NMN mouse sperm; CA, control mouse sperm treated with AA; NA, NMN mouse sperm treated with AA; CF, control mouse sperm treated with FCCP; NF, NMN mouse sperm treated with FCCP. Data are shown as mean ± s.e.m., *n* = 10–13 per group. Black bars are NMN-treated groups. Data were analysed using two-way ANOVA. Note Fig. 3A and B are measurements from the same sperm aliquot. Figure 3C and D are measurements of a different sperm aliquot, but from the same mice as for Fig. 3A and B. Data are mean ± s.e.m.

We examined live sperm with a FACS assay that measured incorporation of the dye 7-AAD as an indicator of viability. However, NMN had no significant effect on viability (Fig. 3A). To measure sperm oxidative stress, and MMP, we treated sperm with MitoSOX Red and JC-1 dyes, respectively. As positive controls, we treated sperm with arachidonic acid to increase sperm oxidative stress and FCCP to reduce MMP. Both arachidonic acid (AA) and carbonyl cyanide-trifluoromethoxyphenylhydrazone (FCCP) reduced viability in both tests (*P* ≤ 0.001) (Fig. 3A and C). Unexpectedly, the percentage of live sperm that is low 7-AAD staining was lower in the sperm used in the oxidative stress assay (that were stained with 7-AAD and MSR) than the sperm used to investigate MMP (stained with 7-AAD and JC-1) (Fig. 2A vs C), despite the experiments using aliquots of sperm from the same mice. This could be due to the MSR and JC-1 stains themselves having differential impacts on sperm viability or on the intensity of 7-AAD fluorescence. However, as the experimental groups were balanced these unexpected effects did not obstruct the investigation of the effects of NMN.

As expected AA significantly increased high MSR levels (*P* = 0.003) (Fig. 3B). However, there was no significant effect of NMN on high MSR levels (Fig. 3B). Looking at MMP, two separate two-way ANOVAs were performed to look at the effect of NMN on HO levels and its effect on HG levels. JC-1 is predominantly a monomer at low MMP and yields green fluorescence. At high MMP, JC-1 aggregates and yields an orange emission. Therefore, the fluorescence reflects the amount of mitochondria that are in a state of high or low MMP. As expected of a mitochondrial uncoupler, FCCP significantly decreased sperm viability (*P* ≤ 0.001) (Fig. 3C). Analysis revealed that there was no significant effect of NMN on HO levels. However, there was a significant reduction of HG level when treated with NMN (*P* = 0.037) (Fig. 3C).

#### Measurement of testes NAD^+^ and NADH levels

**Figure 4:**
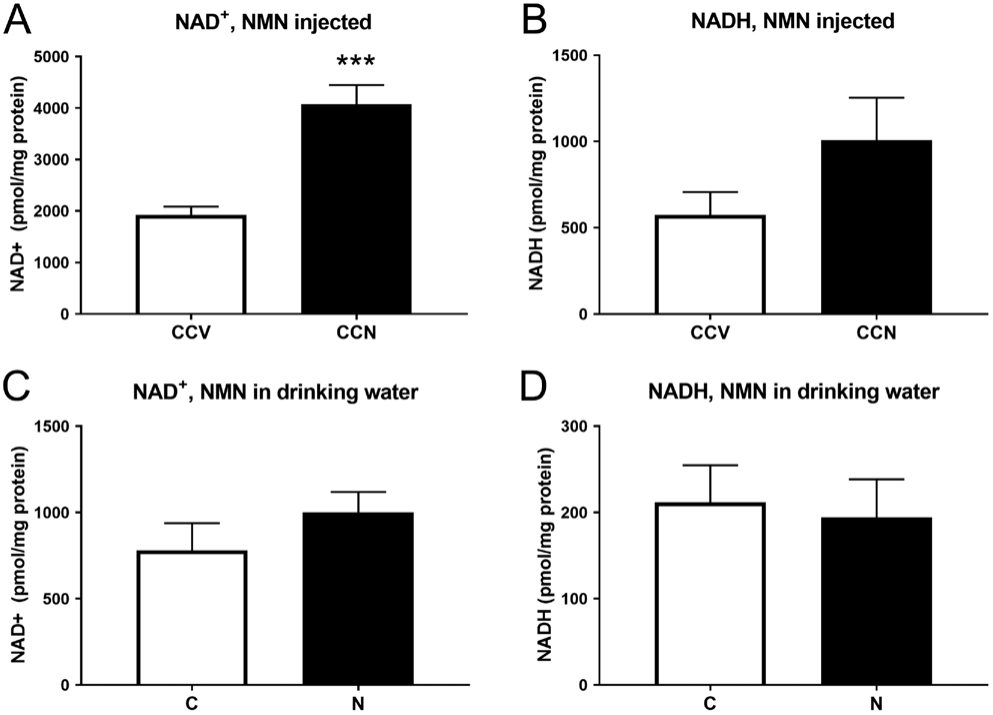
NAD^+^ and NADH levels in testes of mice from both cohorts (NMN injected and NMN in drinking water). Daily intraperitoneal injection of 500 mg/kg body weight NMN (A) increased NAD^+^ levels in testes (***P = 5 × 10^−5^) while (B) NADH levels had a trend for increase (*P* = 0.15). Daily consumption 400 mg/kg body weight NMN in drinking water did not increase (C) NAD^+^ or (D) NADH in testes. Data are mean ± s.e.m., *n* = 9/10 per group in the i.p. cohort and 5/10 in the drinking water cohort.

NAD^+^ levels were doubled in the testes of mice that received i.p. NMN injections compared to those receiving vehicle PBS (Fig. 4A, *P* = 5 × 10^−5^). There was also a trend for NADH to be increased (Fig. 4B, *P* = 0.15). However, no differences in NAD^+^ or NADH were seen in the testes of mice receiving NMN in drinking water (Fig. 4C and D).

## Discussion

Our maternal obesity cohort allowed examination of the effects of two periods of HFD exposure (‘maternal’ i.e. while *in utero* and during lactation, and post-weaning) on widely used indicators of sperm quality. Additionally, effects of the NAD^+^ boosting drug, NMN, on sperm quality were examined. As far as we are aware, no previous studies have compared the effects of diet-induced obesity, maternal obesity and their combination on sperm quality.

The effects on offspring body weights were profound with the expected increases observed due to both maternal HFD and post-weaning HFD. The combination of HFD during the two time periods exacerbated the effect on body weight, so that the mice with the greatest body weights were the HH groups (a doubling in body weight compared to the CC groups), as has also been observed in earlier studies (Bahari *et al.* 2013, Uddin *et al.* 2017). A reduction in sperm count and motility due to post-weaning HFD mirrors the situation observed in many human obesity studies (reviewed in Craig *et al.* 2017). Similarly, the minor effects of post-weaning HFD on sperm DNA and chromatin integrity in our experiment (only a trend in %DFI, *P* = 0.06) may also recapitulate the human situation, as some studies have found associations between high BMI and high DFI (Kort *et al.* 2006), but others have not (Bandel *et al.* 2015, Oliveira *et al.* 2018). The reduction in mtDNA copy number due to post-weaning HFD may be an indicator of sperm mitochondrial dysfunction (Malik & Czajka 2013, Oliveira *et al.* 2018), although mouse experiments have shown that fertility can be maintained even with a genetically induced threefold reduction in mtDNA (Wai *et al.* 2010).

Few studies in humans and animals have examined the relationship between maternal obesity and male offspring sperm quality. Two studies from members of the same group (Rodriguez-Gonzalez *et al.* 2015, Santos *et al.* 2015) showed that male pups of obese rat dams had reduced fertility and poor sperm quality, based on sperm parameters (reduced concentration, % viability and % motility) compared to male pups of non-obese dams. They also observed several changes that highlighted increases in offspring sperm oxidative stress due to maternal obesity. Even though we detected increased CMA3 and SCSA DFI, indicating that maternal obesity impacted sperm chromatin (Supplementary Table 2), we did not observe changes to sperm concentration, viability and motility, nor an increase in the oxidative stress marker 8-OHdG. Additionally, the brothers of the mice used in this study were mated to enable an examination of the F2 generation (unpublished), but no changes in fertility (as determined by number of litters sired and average pups per litter) were seen (data not shown). These differences are likely to be due to species- and diet-specific impacts of maternal obesity or possibly the extent of weight gain achieved in this study.

In the two rat studies by Santos *et al.* (2015) and Rodriguez-Gonzalez *et al.* (2015), the male pups of obese and control dams received only chow diet post-weaning so only had high levels of adiposity when their rats were aged to nearly 2 years. Interestingly, in those studies, the impacts on sperm oxidative stress, concentration, viability and even fertility preceded the age-associated extreme adiposity suggesting that the negative effects of maternal obesity on male offspring reproduction may not be dependent on obesity. In support of this, in our study, it was interesting to note that while the body weight (Fig. 2A) and adiposity of the mice was exacerbated by the combination of maternal- and post-weaning HFD, there were no obvious additive effects in any of the sperm parameters. This may suggest that the effects of maternal obesity occur primarily through alteration of early developmental processes, in prospermatogonia for example, while obesity-induced post-weaning affects the later stages of spermatogenesis (Christante *et al.* 2013).

The only human study to date on the effects of maternal obesity on offspring sperm had a relatively small sample size (*n* = 25 sons of mothers with pre-pregnant BMI >25 vs *n* = 265 from mothers with BMI 18.5–24.99) (Ramlau-Hansen *et al.* 2007). Trends were observed for these sons to have reduced sperm concentration, percent motile sperm and serum testosterone. A larger cohort is clearly needed to properly understand the effects of maternal obesity on male offspring fertility and sperm quality in humans. One potential consideration in human studies that examine associations between high BMI in men and their sperm parameters is that maternal BMI is generally not taken into account (and often these data are not available). The shared environment (e.g. social status, diet and lifestyle) and genetic similarity between mother and son will often mean that not only are the males being assessed obese, but they may have been exposed to increased maternal adiposity during gestation.

The effect of NMN administration on sperm has not been previously investigated. With energy being essential for sperm function, especially motility, these cells are highly reliant on the mitochondria. Obese men have been shown to have a reduction in sperm mitochondrial activity (Fariello *et al.* 2012). Therefore, as NMN supplementation can increase mitochondrial biogenesis and function, we hypothesised that sperm quality could be improved in obese mice. As expected (Uddin *et al.* 2016, 2017), in this experiment, NMN administration to mice fed a HFD reduced body weight, especially in the heaviest mice. However, we also found that NMN reduced sperm count and vitality and increased 8-OHdG.

One explanation for the increased oxidative stress (as indicated by 8-OHdG) was that the NMN administration was increasing ROS. We considered two mechanisms through which NMN could increase sperm ROS. Firstly, we and others have shown that NMN administration increases mitochondrial biogenesis and function (Gomes *et al.* 2013, Uddin *et al.* 2016, 2017). The mitochondria convert NADH to NAD^+^ through a series of processes in energy metabolism and a by-product of this conversion is ROS, therefore, an increase in mitochondrial activity could increase ROS and oxidative stress. The NADH-dependent oxidoreductase reaction in mitochondria is the main source of ROS in sperm so potentially perturbation of NADH levels by NMN administration could increase mitochondrial ROS output. In support of this possibility, measurement of sperm mtDNA copy number in the mice showed an interaction between maternal diet and NMN. MtDNA copy number was increased in offspring from a chow-fed mother, but decreased in offspring from a HFD-fed mother. However, it is unknown whether both of these effects could result in similar increases in 8-OHdG. Secondly, ROS production from sperm plasma membranes is caused by NADPH oxidase (Shukla *et al.* 2005). Through a short biochemical pathway NMN could increase superoxide that is NMN to NADH (by NMNAT2), to NADPH (by NADH kinase) to superoxide (by NADPH oxidase).

Another possible mechanism that could explain the negative effects of NMN administration is that it could increase nicotinamide (NAM) levels as has been seen in mice with supplementation of another NAD^+^-precursor, nicotinamide riboside (NR) (Frederick *et al.* 2016). NAM is known to inhibit sirtuins (Bitterman *et al.* 2002) which are required for normal spermatogenesis (Coussens *et al.* 2008). However, this may contrast with a study that associated long-term supplementation with the sirtuin-activating drug resveratrol with increased mouse sperm DNA damage and 8-OHdG levels (Katen *et al.* 2016). It will be interesting to determine if other NAD^+^ precursors such as nicotinic acid riboside (NAR), nicotinic acid mononucleotide (NaMN) and NR have negative effect on sperm quality at high levels.

To investigate whether NMN administration itself has negative impacts on sperm parameters, we examined a second cohort, independent of obesity in mice only fed regular chow. In this experiment, NMN was administered through drinking water at a slightly reduced dose (chronic rather than acute), which has nonetheless been shown to have beneficial effects on improving muscle blood vessel density and endurance in mice (Das *et al.* 2018). Unlike the maternal obesity experiment no differences in sperm count, viability or sperm mtDNA copy number were seen in these mice. Additionally, the FACS assays on live sperm showed no evidence for alterations to sperm oxidative stress or mitochondrial dysfunction. The only effect of NMN was a reduction in the HG proportion of viable sperm in the JC-1 MMP assay. However, the ratio between HO and HG fluorescence was unaffected by NMN (Supplementary Fig. 2). In summary, minimal effects of NMN on sperm characteristics were observed under these conditions.

This oral administration cohort therefore suggests that chronic NMN administration can have beneficial effects on angiogenesis (Das *et al.* 2018) without impacting sperm quality. It may be that the effects of NMN on sperm quality, as assessed by 8-OHdG, seen in the maternal obesity cohort relate to the surge in NMN following acute injection versus gradual ingestion over the darkness phase (i.e. through the night), although further work would be required to test this. In particular, a future study investigating a group of mice receiving NMN through i.p. injection and another group receiving the same dose in drinking water would be enlightening.

Potential explanations for the differences in sperm states between the maternal obesity (i.p. injected NMN) cohort and the oral administration cohort may lie in the NAD^+^ and NADH levels observed in the testes of representatives of each cohort (Fig. 4). These experiments showed large increases in NAD^+^ and potentially NADH in the testes of the mice that had high sperm ROS, as well as low sperm count and vitality. These data support the possibility that increases in NAD^+^ and/or NADH could alter ROS production through one, or several, of the mechanisms mentioned above, NADH-dependent oxidoreductase reactions, NADPH oxidases and Sirtuin-mediated reactions. These precise mechanistic explanations for how i.p. NMN administration could cause sperm oxidative stress could in future be investigated through mass-spectrometry of testes or sperm for metabolites such as NAM, NADPH and NADH. Additionally, live sperm JC-1 staining in sperm from mice that have had i.p. NMN administration could reveal if the sperm deficits (vitality and 8-OHdG) are associated with altered mitochondrial activity.

## Conclusions

Our data suggest that both maternal obesity and post-weaning diet-induced obesity are risk factors for reduced sperm quality. However, ways in which these two exposures impact sperm may differ and are not additive. Our examination of the effects of NMN supplementation point to dose- and/or route-of-administration differences in the consequences for sperm quality, which invites further investigation.

## Supplementary Material

Supplementary figure 1. Process of analysing flow cytometry data in Oral Administration Cohort. Cytograms and histograms representing A) side-scatter vs. forward-scatter cytogram gated for sperm cells, B) 7AAD vs. SSC cytogram gated for live cells, C) histogram for 7AAD vs. count with a border set to determine 7AAD negative and 7AAD positive sperm cells, D) phycoerythrin (PE) vs. SSC cytogram gated for low and high MSR live sperm cells, E) histogram for PE vs. count with a border set to determine low MSR cells and high MSR cells, and F) fluorescein isothiocyanate (FITC) vs. PE scatterplot divided into four quadrants: Q1 with low FITC high PE (LFHP), Q2 with high FITC high PE-A (HFHP), Q3 with high FITC low PE (HFLP), and Q4 with low FITC low PE (LFLP).

Supplementary Figure 2. Mitochondrial membrane potential of sperm from Oral Administration Cohort – Ratio of High Orange to High Green. J-C (JC-1+7-AAD), J-N (JC-1+7-AAD+NMN), JF-C (JC-1+7-AAD+FCCP), J-N (JC-1+7-AAD+NMN+FCCP). Data are shown as mean±SEM, n=10-13 per group. Data were analysed using two-way ANOVA. Overall main effects are indicated by a horizontal bar above groups: ††† p ≤ 0.001 FCCP effect.

Supplementary table 1. Values and concentrations of tube contents for mouse FACS experiments (Oral Administration Cohort). To assess MMP in viable sperm cells JC-1 and 7-AAD was added into the sperm aliquot. Carbonyl cyanide-trifluoromethoxyphenylhydrazone (FCCP) was added as a positive control. DMSO was added as vehicle.

Supplementary table 2. Effects of maternal obesity, offspring HFD and intraperitoneal NMN treatment on anthropometric and sperm parameters in mouse offspring, Maternal Obesity Cohort. The top section displays the group mean and SEM parameter for various parameters. The bottom section of the table displays P values from a three way ANOVA that was used to compare all 8 groups where maternal diet, post-weaning diet and treatment were three different factors. The first letter of the group code indicates maternal diet, chow (C) or HFD (H). The second letter indicates offspring post-weaning diet, chow (C) or HFD (H). The third letter indicates NMN (N) or vehicle (V) injection.

Supplementary table 3. Effects of mouse oral NMN treatment on anthropometric and sperm parameters (Oral Administration Cohort).

## Declaration of interest

D A S is a founder, equity owner, board member, advisor to, director of, consultant to, investor in and/or inventor on patents licensed to Vium, Jupiter Orphan Therapeutics, Cohbar, Galilei Biosciences, GlaxoSmithKline, OvaScience, EMD Millipore, Wellomics, Inside Tracker, Caudalie, Bayer Crop Science, Longwood Fund, Zymo Research, EdenRoc Sciences (and affiliates Arc-Bio, Dovetail Genomics, Claret Bioscience, Revere Biosensors, UpRNA and MetroBiotech (an NAD booster company), Liberty Biosecurity), Life Biosciences (and affiliates Selphagy, Senolytic Therapeutics, Spotlight Biosciences, Animal Biosciences, Iduna, Immetas, Prana, Continuum Biosciences, Jumpstart Fertility (an NAD booster company), and Lua Communications). D A S sits on the board of directors of both companies. D A S is an inventor on a patent application filed by Mayo Clinic and Harvard Medical School that has been licensed to Elysium Health; his personal royalty share is directed to the Sinclair lab. For more information see https://genetics.med.harvard.edu/sinclair-test/people/sinclair-other.php. Prof. R John Aitken is on the editorial board of *Reproduction*. Prof. Aitken was not involved in the review or editorial process for this paper, on which he is listed as an author. The other authors have nothing to disclose.

## Funding

This research was funded by National Health and Medical Research Council (NHMRC) project grant #1044295 to M J M and D A S. G M U was supported by a University International Postgraduate Award (UIPA), UNSW Sydney. DAS was supported by The Glenn Foundation for Medical Research, and NIH R01AG028730 / R01DK100263

## Author contribution statement

M J M, D A S, N A Y, R J A and A D contributed to experimental conception and design. G M U, N A Y, C M, A D, C M, J Y, H S C and S W performed the experiments. N A Y, G M U, C M and M J M analysed the data. N A Y, C M and M J M wrote the first draft of the paper. All authors reviewed and approved the final manuscript.
