# Supplementary figures and images for: Impacts of obesity, maternal obesity and nicotinamide mononucleotide supplementation on sperm quality in mice
Source: Reproduction. 2019 May 30;158(2):171–81. doi: 10.1530/REP-18-0574 (PMC6589912; doi:10.1530/REP-18-0574)

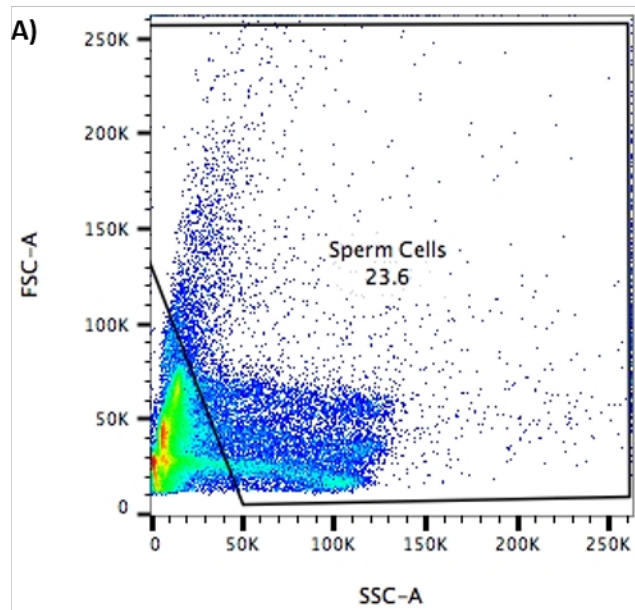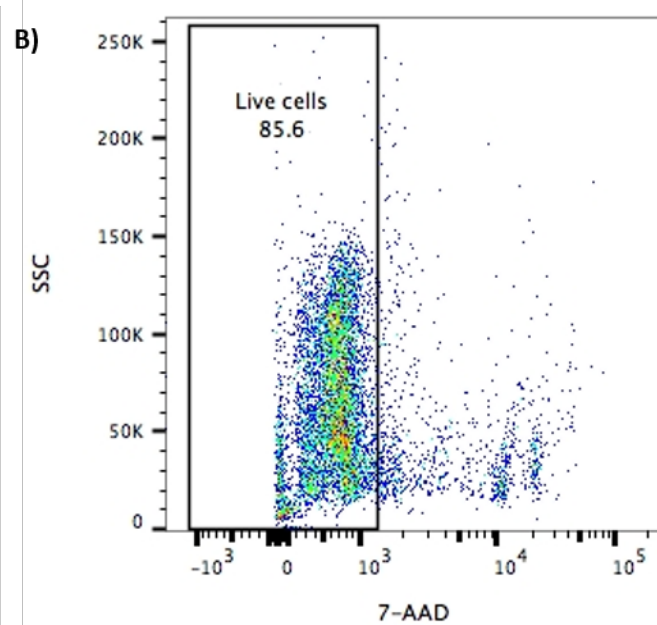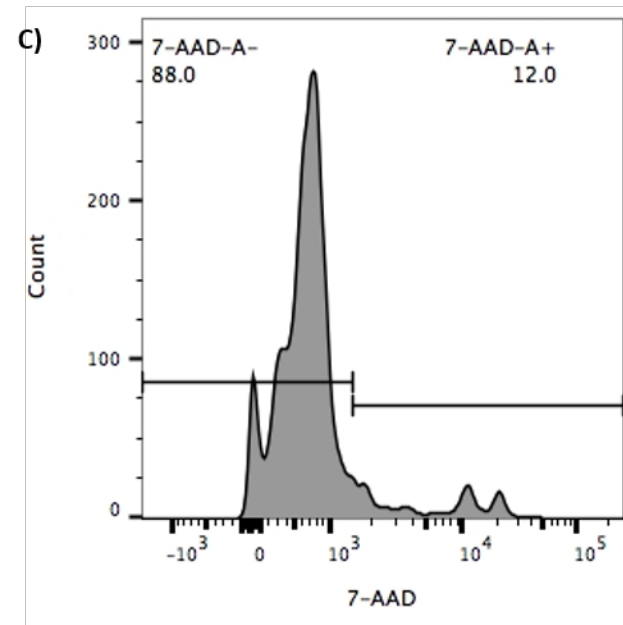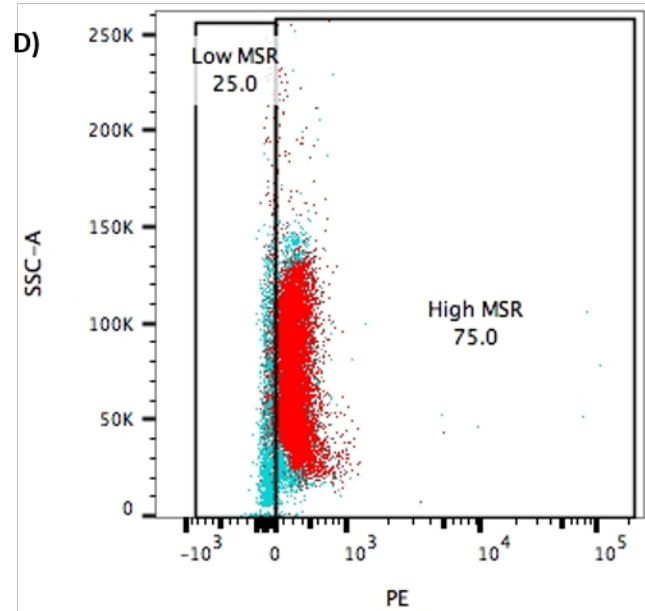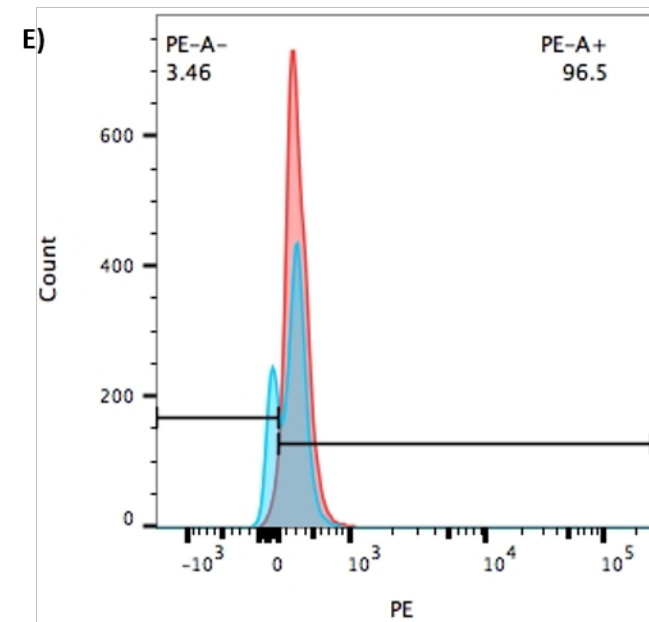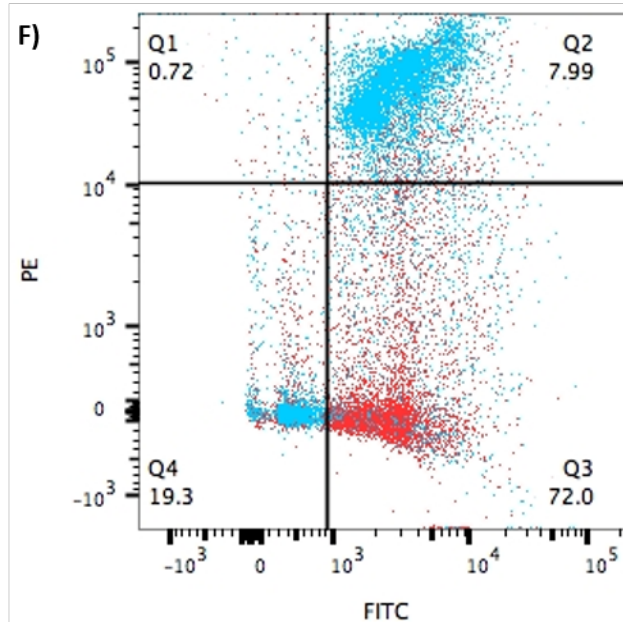

Supplement: Supplementary figure 1. Process of analysing flow cytometry data in Oral Administration Cohort. Cytograms and histograms representing A) side-scatter vs. forward-scatter cytogram gated for sperm cells, B) 7AAD vs. SSC cytogram gated for live cells, C) histogram for 7AAD vs. count with a border set t [file supplementary_figure_1.pdf]

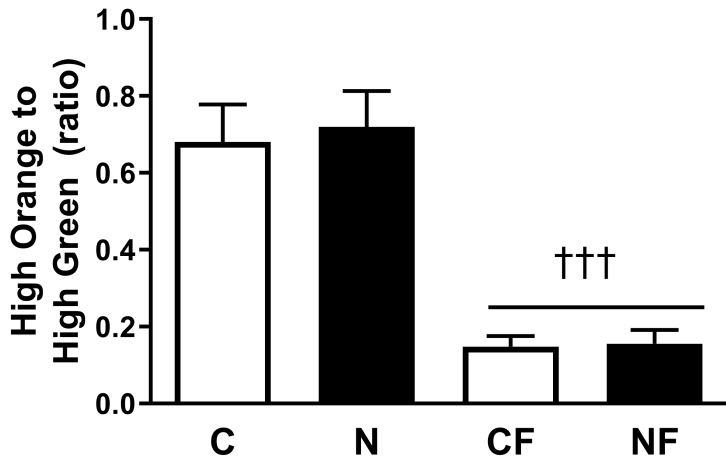

Supplement: Supplementary Figure 2. Mitochondrial membrane potential of sperm from Oral Administration Cohort – Ratio of High Orange to High Green. J-C (JC-1+7-AAD), J-N (JC-1+7-AAD+NMN), JF-C (JC-1+7-AAD+FCCP), J-N (JC-1+7-AAD+NMN+FCCP). Data are shown as mean±SEM, n=10-13 per group. Data were analysed using t [file supplementary_figure_2.pdf]
