# Supplementary material for: Impacts of obesity, maternal obesity and nicotinamide mononucleotide supplementation on sperm quality in mice
Source: Reproduction. 2019 May 30;158(2):171–81. doi: 10.1530/REP-18-0574 (PMC6589912; doi:10.1530/REP-18-0574)
Supplement: Supplementary table 1. Values and concentrations of tube contents for mouse FACS experiments (Oral Administration Cohort). To assess MMP in viable sperm cells JC-1 and 7-AAD was added into the sperm aliquot. Carbonyl cyanide-trifluoromethoxyphenylhydrazone (FCCP) was added as a positive control. DMS [file supplementary_table_1.pdf]

| Mitochondrial membrane potential | | |
|---|---|---|
| Components | **Test (J7)** | **Positive control (J7F)** |
| Sperm aliquot ($10^6$/mL) | 190µL | 190µL |
| 7-AAD (concentration is proprietary) | 4µL | 4µL |
| JC-1 (1mM) | 4µL | 4µL |
| FCCP (10mM) in DMSO | - | 1µL |
| DMSO | 1µL | - |

| Oxidative Stress | | |
|---|---|---|
| Components | **Test (M7)** | **Positive control (M7AA)** |
| Sperm aliquot ($10^6$/mL) | 190µL | 190µL |
| 7-AAD (concentration is proprietary) | 4µL | 4µL |
| MSR (50µg in 13µL DMSO) | 4µL | 4µL |
| AA (50mM) in EtOH | - | 4µL |
| Ethanol | 4µL | - |

**Supplementary table 1. Values and concentrations of tube contents for mouse FACS experiments (Oral Administration Cohort).** To assess MMP in viable sperm cells JC-1 and 7-AAD was added into the sperm aliquot. Carbonyl cyanide-trifluoromethoxyphenylhydrazone (FCCP) was added as a positive control. DMSO was added as vehicle.

To assess oxidative stress in live sperm cells, MSR and 7-AAD were added into the sperm aliquot. Arachidonic acid (AA) was added as a positive control. Ethanol was added as vehicle.
