# Supplementary material for: Impacts of obesity, maternal obesity and nicotinamide mononucleotide supplementation on sperm quality in mice
Source: Reproduction. 2019 May 30;158(2):171–81. doi: 10.1530/REP-18-0574 (PMC6589912; doi:10.1530/REP-18-0574)
Supplement: Supplementary table 2. Effects of maternal obesity, offspring HFD and intraperitoneal NMN treatment on anthropometric and sperm parameters in mouse offspring, Maternal Obesity Cohort. The top section displays the group mean and SEM parameter for various parameters. The bottom section of the table di [file supplementary_table_2.pdf]

Treatment Group	CCV	CCN	CHV	CHN	HCV	HCN	HHV	HHN
Body length (cm) n=5-12	9.00±0.08	9.12±0.06	9.61±0.07	9.63±0.08	9.13±0.09	9.08±0.09	9.78±0.08	9.51±0.10
Testis weight / body length n=5-12	0.0197±0.0005	0.0202±0.0005	0.0197±0.0007	0.0198±0.0003	0.0217±0.0005	0.0222±0.0003	0.0206±0.0006	0.0216±0.0005
mtDNA copy number relative to average of CCV n=9-12	1.00±0.12	1.25±0.12	0.77±0.08	0.82±0.08	1.14±0.09	0.88±0.06	0.82±0.06	0.67±0.06
Halo (%) n=6-11	37.3±16.7	33.6±14.7	22.7±10.8	32.4±11.7	61.0±19.8	36.8±11.6	39.0±12.9	22.7±11.0
CMA3 (%) n=6-11	2.2±0.8	2.9±0.8	2.9±0.4	2.1±0.5	4.8±1.6	3.6±0.9	3.3±0.9	4.2±0.8
SCSA (% DFI) n=6-11	29.2±0.8	29.0±0.5	29.5±0.8	30.2±0.8	30.1±0.6	29.8±0.5	30.7±0.8	31.9±0.9

	**3-way ANOVA outcome**					
**Treatment Group**	**Main effects**			**Interactions**		
	Maternal diet	Post-weaning diet	NMN	Mat Diet * Diet	Mat Diet * NMN	Diet * NMN
Body length (cm) n=5-12		4x10-11				
Testis weight / body length n=5-12	<0.0001					
mtDNA copy number relative to average of CCV n=9-12		<0.0001			<0.01	
Halo (%) n=6-11						
CMA3 (%) n=6-11	<0.01					
SCSA (% DFI) n=6-11	<0.05	0.06				

Supplementary table 2. Effects of maternal obesity, offspring HFD and intraperitoneal NMN treatment on anthropometric and sperm parameters in mouse offspring, Maternal Obesity Cohort. The top section displays the group mean and SEM parameter for various parameters. The bottom section of the table displays P values from a three way ANOVA that was used to compare all 8 groups where maternal diet, post-weaning diet and treatment were three different factors. The first letter of the group code indicates maternal diet, chow (C) or HFD (H). The second letter indicates offspring post-weaning diet, chow (C) or HFD (H). The third letter indicates NMN (N) or vehicle (V) injection.
