# Supplementary material for: Impacts of obesity, maternal obesity and nicotinamide mononucleotide supplementation on sperm quality in mice
Source: Reproduction. 2019 May 30;158(2):171–81. doi: 10.1530/REP-18-0574 (PMC6589912; doi:10.1530/REP-18-0574)
Supplement: Supplementary table 3. Effects of mouse oral NMN treatment on anthropometric and sperm parameters (Oral Administration Cohort). [file supplementary_table_3.pdf]

**Treatment Group**	Vehicle	NMN	T-test V vs N
Body weight (g) n=11-13	35.7±1.0 min 30.6 max 42.0	36.2±1.0 min 30.1 max 40.6	P=0.28
Sperm count (per ml x10^5) n=11-13	44.4±3.2 min 28.0 max 60.0	46.8±3.6 min 25.0 max 65.0	P=0.23
Motile (%) n=9-10	25.6±3.3 min 6.7 max 40.0	13.0±3.5 min 1.0 max 35.0	P=0.13
mtDNA copy number relative to average of Vehicle n=11-12	1±0.06 min 0.8 max 1.3	0.94±0.02 min 0.8 max 1.0	P=0.51

Supplementary table 3. Effects of mouse oral NMN treatment on anthropometric and sperm parameters (Oral Administration Cohort).
